# Diagnostic Accuracy of Lung Ultrasound in Neonatal Diseases: A Systematized Review

**DOI:** 10.3390/jcm13113107

**Published:** 2024-05-25

**Authors:** Stefano Nobile, Lucia Sette, Claudia Esposito, Francesca Riitano, Chiara Di Sipio Morgia, Annamaria Sbordone, Giovanni Vento, Alessandro Perri

**Affiliations:** 1 Neonatal Unit, Department of Mother, Child and Public Health, Fondazione Policlinico Universitario A. Gemelli IRCCS, Largo F. Vito 1, 00168 Rome, Italy; 2 Department of Woman, Child, General and Specialistic Surgery, University of Campania “Luigi Vanvitelli”, 80138 Naples, Italy; claudia.esposito3@studenti.unicampania.it

**Keywords:** thoracic ultrasound, infants, neonatal intensive care unit, respiratory distress, chronic lung disease

## Abstract

**Background:** Respiratory problems are frequent in newborns, and are mainly studied with chest X-rays, whereas CT scans are usually needed for the evaluation of rare malformations and diseases. Lung ultrasound (LUS] has been proposed as an alternative method of diagnosing a variety of respiratory conditions. In recent years, there has been a rapid increase in LUS studies, thanks to the ability of LUS to rapidly exclude complications and significantly reduce radiation exposure in this fragile population. We aimed to summarize the current knowledge about LUS. **Methods:** A literature search was conducted on the Medline and Cochrane databases using appropriate terms. The inclusion criteria were: English language and human species. Exclusion criteria were: non-English language, animal species, case reports, case series, non-systematic reviews, and editorials. **Results:** The search returned 360 results. No Cochrane reviews were found. Titles and abstracts were screened, and 37 were finally considered. Studies concerning the use of lung ultrasound for the following conditions were presented: neonatal respiratory distress syndrome, transient tachypnea of the newborn, pneumothorax, pulmonary hemorrhage, pneumonia, bronchopulmonary dysplasia, and prediction of extubation success. **Conclusions:** We discussed the utility of LUS for the diagnosis and treatment of neonatal diseases according to the most recent literature.

## 1. Introduction

Respiratory problems are among the most frequent reasons for admission to neonatal units. Evidence-based practice has influenced several changes in recent years, including the implementation of antenatal steroid administration and non-invasive ventilation, the optimization of surfactant administration, and caffeine administration [1]. Around 10% of infants are born preterm and prone to respiratory distress syndrome (RDS]. Evidence suggests that RDS incidence is inversely associated with gestational age (GA] at birth [1,2]. In fact, alveolarization begins around 36 weeks’ GA [3]. Extremely preterm infants are born during the early phases of lung development (<28 weeks’ GA), when the lung surface area is still limited and immature, even if some degree of gas exchange is possible. Moreover, recent studies reported an increased incidence of chronic lung disease (bronchopulmonary dysplasia, BPD) even in infants without severe RDS, most likely due to the increased survival of preterm infants [4,5]. BPD affects 18% of very low-birth-weight (<1500 g) infants in Europe [1]. 

Respiratory diseases in infants are mainly studied with chest X-rays (CXR), whereas CT scans are usually needed for the evaluation of rare malformations and diseases. Until the 1990s physicians thought that the lung was unexplorable by ultrasound, as air and bones stopped the propagation of the ultrasound beam [6]. However, the pioneering research by Lichtenstein and colleagues [6] revealed the significance of reverberation artefacts behind the pleural line and other ultrasound images.

Since then, lung ultrasound (LUS) has been proposed as an alternative method of diagnosing a variety of respiratory conditions. In neonatology, in particular, there has been a rapid increase in LUS studies, thanks to the ability of this method to rapidly exclude complications (i.e., pneumothorax, PTX) and to significantly reduce radiation exposure in this fragile population [7,8]. Considering the long life expectancy of preterm infants, perinatal exposures (including radiation) and conditions may influence the development of later (paediatric and adult) diseases, as proposed by the Developmental Origins of Health and Disease Hypothesis [9].

Potential benefits of LUS compared to CXR, as previously reported [7,8,10,11], include the reduction of radiation exposure to infants, faster recognition of critical diseases (i.e., PTX), and potentially better diagnostic accuracy and guidance for therapy (i.e., surfactant for RDS).

Potential disadvantages include local availability of ultrasound machines and probes, costs, the need for training, the potential contrast with minimal handling practices in preterm infants, quality assurance, and the potential difficult initial learning process (i.e., the so-called “steep learning curve”).

The purpose of this work is to systematically review the utility of lung ultrasound in the most frequent neonatal diseases. A quality assessment of the included studies and a narrative and synthesis of the evidence are presented.

## 2. Materials and Methods

A literature search was conducted on the Medline and Cochrane databases. The publication date was from inception to the last update (performed on 1 March 2024). Search terms were: “lung ultrasound”, “newborn”, “infant”, “preterm infant”, “respiratory distress syndrome”, “transient tachypn?ea of the newborn”, “pneumothorax”, bronchopulmonary dysplasia”, “chronic lung disease”, “pneumonia”, “meconium aspiration syndrome”, “pulmonary h?emorrhage”, “radiation exposure”; the MeSH terms “infant, premature”, “respiratory distress syndrome, newborn”, “transient tachypnea of the newborn”, “pneumothorax”, “bronchopulmonary dysplasia”, “pneumonia”, “meconium aspiration syndrome”. Their combinations were explored using the Boolean operator “AND” as appropriate. The inclusion criteria were the English language and human species. Exclusion criteria were: non-English language, animal species, case reports, case series, non-systematic reviews, and editorials. The protocol was not registered as it was not a systematic review.

## 3. Results

### 3.1. Literature Search

**Figure 1:**
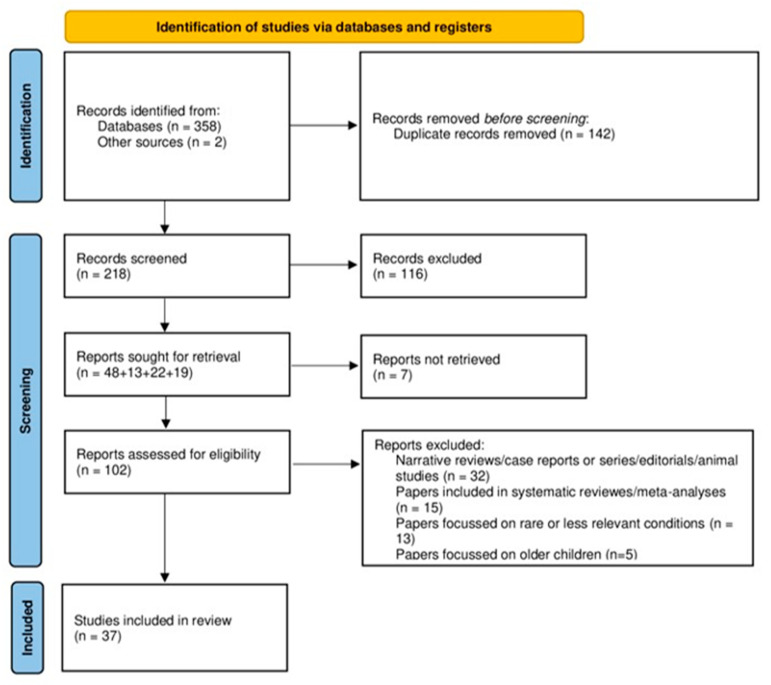
Audit trail of the evidence (PRISMA flow diagram).

The search returned 360 results. No Cochrane reviews were found. Titles and abstracts were screened, and 37 were finally considered. An audit trail of the evidence is shown in Figure 1.

### 3.2. Critical Appraisal of the Evidence

RDS is mainly due to surfactant deficiency and results in pulmonary insufficiency soon after birth, particularly in preterm infants [1]. Clinical description and management have evolved in recent years due to the improvement of neonatal care (i.e., preventive, diagnostic, and therapeutic tools). Recommendations for prenatal care, delivery room stabilization, respiratory support, surfactant therapy, and supportive care are periodically updated by a panel of European neonatologists. In particular, early identification and treatment of RDS may reduce the risk of death and BPD [12,13].

The diagnosis of RDS is based on the clinical status of patients in correlation with laboratory parameters and chest X-rays. Lung ultrasound, even if widely used, is still not incorporated into diagnostic algorithms [11].

European data from 2014 to 2016 show that around 50% of all babies born between 22 + 0 and 32 + 6 weeks receive surfactant; therefore, recognizing the optimal timing and indication for surfactant administration is important, particularly because infants treated with surfactant therapy within 2–3 h of life are at lower risk of death and BPD compared with infants treated later [1].

Ma et al. [14] conducted a systematic review and meta-analysis (9 studies, 703 patients) to calculate the pooled sensitivity and specificity of LUS in diagnosing RDS (with a combination of clinical signs and symptoms, CXR with/without laboratory blood gas analysis) and evaluate its diagnostic accuracy. Significant heterogeneity between the included studies regarded several features, including variable LUS methodology (abdominal versus thoracic approach), time interval between LUS and CXR, study design (cohort versus case–control), and diagnostic criteria for RDS (combinations of clinical, CXR, and laboratory aspects). Therefore, the authors performed subgroup analyses. For the six studies that used transthoracic scanning, LUS sensitivity was 99% (95% CI: 96–100%) and specificity was 97% (both higher than the transabdominal approach), compared to 91% and 84% for CXR. The main issue for this kind of study is that diagnostic criteria for RDS are not straightforward and evolve with time, as indicated by the periodic consensus guidelines by Sweet et al. [1]. The generalizability and validity of the results are limited because eligible studies included infants born at different gestational ages, with potential differences in clinical characteristics; also, there was some heterogeneity in LUS techniques and equipment.

A systematic review and meta-analysis by Razak et al. [10] was performed to assess LUS accuracy in determining the need for surfactant treatment or mechanical ventilation in infants with RDS treated with nasal continuous positive airway pressure (NCPAP). The authors included six studies (485 infants), three of which used the LUS score. The pooled sensitivity and specificity at LUS score cutoffs > 5–6 were 88% (95% CI 80–93%) and 82% (95% CI 74–89%), respectively. Infants with LUS scores > 5–6 were at significantly increased risk of surfactant treatment compared with infants with LUS scores < 5–6 (relative risk = 7.51; 95% CI 4.16 to 13.58; two studies, 189 participants). A LUS score > 10 showed 84% sensitivity and 70% specificity for surfactant retreatment (one study). The results are reliable since the meta-analysis included studies with similar models (LUS score) and preterm infants born < 34 weeks of gestation, resulting in low heterogeneity. The main limitation for the generalizability of the results is that the calculation of the LUS score might be influenced by multiple factors (i.e., probe, technique, operator skills).

Different studies were run to assess the predictive role of LUS to predict and guide surfactant administration. Luo et al. performed a systematic review of those who investigated the application of lung ultrasound in predicting surfactant use [15]. Ten studies with 1162 participants were included. They found that the sensitivity and specificity of lung ultrasound in predicting surfactant use were 0.86 (95% CI = 0.81–0.90) and 0.82 (95% CI = 0.71–0.90), respectively. The authors also tried to determine an optimal LUS timing and cutoff for predicting surfactant use and concluded that lung ultrasound within 1–3 h after birth and a LUS cutoff of 5 had good performance and sensitivity. The limitations of this review were the differences in patient populations and the small sample size of subgroups, as well as the fact that most but not all of the included studies used the same lung ultrasound diagnostic criteria.

With regard to the diagnostic criteria, Corsini et al. performed an observational, retrospective, multicenter study trying to compare the three main LUS scores proposed by Brat, Raimondi, and Rodriguez-Fanjul to assess if there was one of the three scores, with their respective cutoffs, that had greater ability to predict surfactant need [16]. The cohort of this study included 54 very preterm infants with RDS on non-invasive ventilation with a LU performed prior to surfactant administration (1–3 h of life). Brat, Raimondi, and Rodriguez-Fanjul scores showed a strong ability to predict the need for surfactant: the AUCs were 0.85 (95% CI 0.74–0.96), 0.85 (95% CI 0.75–0.96), and 0.79 (95% CI 0.67–0.92), respectively. No significant differences have been found between the AUCs using the DeLong test. Brat and Raimondi’s scores had an optimal cut-off value > 8, while Rodriguez-Fanjul’s score cutoff was 10. So, even with different cut-off values, all of them had an excellent ability to predict the need for surfactant. Limitations included the retrospective nature of the study and the small sample size.

Perri et al. investigated the use of LUS in the early prediction of CPAP and surfactant among infants born ≥ 33 weeks of gestation [17]. They found a correlation between LUS score and respiratory assistance or need for surfactant administration; also, LUS was significantly associated with the SpO_2_/FIO_2_ ratio, a widely used index of oxygenation, highlighting its role in patient monitoring. In this study, sixty-two patients, with a mean gestational age of 36 weeks, were enrolled. The receiver operating characteristic analysis for the LUS within 3 h of life and at 4–6 h of life yielded an area under the curve of 0.91 and 0.82, respectively, in predicting the need for CPAP. A LUS score cutoff of 6 (sensitivity 84.8%, specificity 86.2%) and 5 (sensitivity 66.7%, specificity 100%) were calculated for LUS performed within 3 h of life and at 4–6 h of life, respectively. The limitations of this study included the observational study design, and the small sample size from just two centers. Furthermore, the LU scans were taken with two different devices, and this may have introduced some heterogeneity.

The value of a combination of oxygen saturation, FIO_2_ ratio, and LUS in predicting surfactant administration was also noticed in an observational multicenter study performed by Raimondi et al. [18]. They enrolled 240 neonates with different gestational ages, of whom 108 received at least one dose of surfactant. LUS predicted the first surfactant administration, without significant difference among different gestational age groups, with an area under the curve (AUC) of 0.86 (95% CI, 0.81–0.91), a cutoff of 9, a sensitivity of 0.79 (95% CI, 0.70–0.86), and a specificity of 0.83 (95% CI, 0.76–0.89), confirming the results of previous studies. The limitations of this work were the fact that oxygenation was investigated only with noninvasive measures and the fact that LUS scans were performed only by expert operators.

Kumar et al. conducted a prospective study on 192 neonates with RDS managed on NIV, aiming to assess the prognostic value of LUS to predict intubation for NIV failure [19]. They found that a LUS score > 7 accurately discriminates between neonates requiring invasive ventilation and those who do not, with a 77.4% sensitivity (95% CI: 58.9–90.8%), 75.1% specificity (95% CI: 67.8–81.7%), and 75.5% overall accuracy (95% CI: 68.8–81.4%) in predicting NIV failure. Furthermore, in this study, the LUS outperformed the Silverman–Andersen score for NIV failure prediction. The limitations of this study include the advanced gestational age of the population, and the observational, single-center design.

Abushady et al. [20] conducted a small, randomized trial to assess LUS efficacy in detecting opening and closing lung pressures during a lung recruitment maneuver in ventilated preterm neonates with RDS and its correlation with pulmonary inflammatory markers. The study was single-blind, included 44 infants (22 for each group), and evaluated short-term outcomes (oxygen and ventilation need, duration of hospital stay, tracheal interleukin-6 levels). The authors found that LUS was useful to attain optimal lung recruitment: LUS-guided recruitment compared to conventional recruitment (based on changes in oxygen need) led to a higher reduction in the fraction of inspired oxygen (FiO_2_), mean airway pressure, PaO_2_/FIO_2_ ratio, lesser oxygen requirements, a shorter duration of ventilation and neonatal intensive care unit (NICU) length of stay, and attenuation of pulmonary inflammatory markers. However, randomization details and clinical data (i.e., antenatal steroid administration, reason for preterm birth, co-morbidities, among others) were not reported, sample size calculation was based on unknown data, and significant outcomes (i.e., mortality, chronic lung disease) were not different between groups. Hence, LUS appears to be a promising tool to guide lung recruitment, but more evidence is needed to assess the long-term clinical benefits of its use. Moreover, recruitment maneuvers are not consistently advised by RDS guidelines and depend on the operator’s skills.

A prospective, multicenter cohort study was performed by Szymański et al. [21] to assess the ability of LUS to predict important outcomes among neonates with RDS. This study included 155 neonates from five centers and evaluated outcomes such as the need for MV at <72 h of life, the need for surfactant administration, successful weaning from NCPAP, extubation readiness, and the development of moderate to severe BPD. Even if the results on extubation readiness were not significant (but the authors did not perform pre-extubation examinations in most patients, and extubation criteria were not stated), the study was adequately powered to detect a significant impact of the LUS in predicting the need for MV at <72 h of life (a cutoff value of 7 was found optimal for prognosis with sensitivity of 89% and specificity of 65%), the need for surfactant (a score of 6 was the best predictive cutoff value with a sensitivity of 88% and specificity 79%), successful weaning from CPAP (in the multivariate model, the LUS score before weaning was the only significant factor influencing weaning success; OR 0.93, 95% CI = 0.90–0.97) and BPD development. Significant factors affecting the occurrence of moderate to severe BPD included the LUS on day 7 (OR = 1.02, 95% CI = 1.00–1.04, *p* = 0.028), gestational age (OR = 0.97, 95% CI = 0.95–0.98, *p* < 0.001), and MV duration (OR = 1.02, 95% CI = 1.00–1.04, *p* = 0.018). The limitations of this study mainly involved the fact that the authors used a BPD definition from 2001; moreover, infants who were primarily intubated (so those who were most vulnerable to developing BPD) were excluded, representing a potential selection bias. Third, the study cohort was largely represented by mature newborns. Finally, the lung scoring system that they used was a modified Brat’s scale, so the generalizability and validity of the results are limited.

Raimondi et al. investigated the utility of lung ultrasound in monitoring the respiratory status of preterm neonates during RDS [22]. They pointed out that LUS has a significant association with major complications (sepsis, patent ductus arteriosus, pneumothorax, and persistent pulmonary hypertension in the neonate). They also found a correlation between LUS score, gestational age, and SpO_2_/FIO_2_ ratio; the strength of the association between LUS and the SpO_2_/FIO_2_ ratio increased with gestational age. Furthermore, they found that LUS is also an early predictor of BPD, especially in the 28 to 30 week gestational age cohort, with an AUC of 0.89, a cutoff of 10, a sensitivity of 0.78, and a specificity of 0.87. A limitation of this study is that it was an observational, single-center study.

TTN is one of the most common causes of neonatal respiratory distress [23]. It is a benign, self-limited condition due to a delay in fetal lung fluid clearing after birth. The incidence of TTN is inversely proportional to gestational age and affects approximately 10% of infants delivered between 33 and 34 weeks and less than 1% of term infants. The traditional diagnosis of TTN is based on clinical characteristics and CXR, even if CXR findings are nonspecific for TTN [23]. Wang et al. [24] conducted a meta-analysis (6 studies, 617 patients) to determine the diagnostic value of LUS for TTN. From an initial pool of 378 articles, nine studies encompassing 3239 patients were included in this meta-analysis. The overall quality of the selected studies ranged from moderate to high. The threshold analysis revealed no discernible threshold effect; however, significant heterogeneity among the studies was observed. Consequently, a random-effects model was employed. The resulting sensitivity, specificity, positive likelihood ratio (PLR), and negative likelihood ratio (NLR) were calculated at 95%, with confidence intervals (CIs) of 0.51–0.58, 0.98–0.99, 14.05–241.88, and 0.18–0.43, respectively. The combined diagnostic odds ratio (DOR) and area under the curve (AUC) were estimated at 95%, with CIs ranging from 68.71 to 6911.79. Subgroup analysis indicated that variations in LUS diagnostic criteria and the choice of gold standard might account for the observed heterogeneity. The pooled sensitivity and specificity for CXR diagnosis were 100% and 99%, respectively. Unlike other organs, lung ultrasound interpretation integrates real anatomical images with artifacts generated by ultrasound beams at the air/fluid interface. Copetti et al. [25] pioneered the utilization of LUS for TTN diagnosis, introducing the concept of “double lung point” to delineate the abrupt transition of echogenicity between lower lung fields exhibiting a hyperechoic, thin pleural line and compact B-lines and the relatively normal upper lung regions. However, the diagnostic efficacy of double lung points for TTN diagnosis has exhibited substantial variability across published studies. Furthermore, the ultrasonic pattern of TTN is subject to variability, with features such as “prevalence of A-lines” in the initial phase and the manifestation of a “white lung” in severe cases. Such discrepancies may confound clinicians and impede the widespread adoption of LUS for TTN diagnosis. Srinivasan et al. [26] conducted a single-center study from January 2020 to June 2021 in order to assess the precision of lung ultrasound for diagnosing and distinguishing between TTN and RDS in preterm neonates. A total of 100 preterm neonates (<37 weeks) with symptoms of respiratory distress within six hours of birth were admitted to the neonatal intensive care unit. Among them, 50 were diagnosed with TTN and 50 with RDS based on a comprehensive clinical examination, laboratory assessments, and chest X-rays. Each neonate underwent a lung ultrasound examination performed by a senior radiologist who remained blinded to the clinical diagnoses. The lung ultrasound findings in both TTN and RDS cases were carefully analyzed and compared. The presence of “double lung point” exhibited 94% sensitivity, 100% specificity, and positive predictive value for diagnosing TTN, while its presence effectively ruled out RDS. However, three cases of severe TTN presented with “white-out” lungs (coalescent B-lines), precluding the visualization of double lung point. Nevertheless, consolidation was not observed in these cases. The presence of consolidation demonstrated a 100% negative predictive value for TTN. The findings of this study underline the accuracy and reliability of lung ultrasound in diagnosing and differentiating between RDS and TTN in preterm neonates. LUS examination was performed by an experienced radiologist, and details about patient recruitment were not provided; hence, the generalizability of the study is somewhat limited.

Pezza et al. conducted a prospective observational study to assess lung aeration and function in neonates using semiquantitative LUS and transcutaneous blood gas measurements conducted at 1 h (time point 0), 6 h (time point 1), 12 h (time point 2), 24 h (time point 3), and 72 h (time point 4) post-birth. Endogenous surfactant levels were estimated through lamellar body count [27]. Primary outcomes included LUS, oxygenation index (OI), oxygen saturation index (OSI), and transcutaneous pressure of carbon dioxide (PtcCO_2_), adjusted for gestational age. Sixty-nine neonates with RDS and 58 with TTN were included. LUS improved over time but consistently indicated worse outcomes for RDS compared to TTN. Oxygenation improved over time but remained poorer for RDS throughout the study period. PtcCO_2_ improved over time and remained similar between the RDS and TTN cohorts. A low lamellar body count was associated with RDS and higher LUS scores. During the initial 72 h post-birth, the RDS cohort consistently exhibited poorer lung function compared to the TTN cohort, whereas CO_2_ clearance remained similar between the groups. Both lung aeration and function improved over time.

Neonatal PTX, an air leak that occurs when air accumulates between the parietal and visceral pleura, is a life-threatening condition with the highest prevalence at ≤24 weeks’ gestational age (GA) and the highest mortality at 29–32 weeks’ GA [28]. Fei et al. [8] performed a systematic review and meta-analysis (8 studies, 529 patients) aimed to compare the diagnostic accuracy of LUS with that of CXR in neonates with PTX. They included prospective studies recruiting preterm infants with respiratory symptoms in which clear LUS aspects of PTX were provided. They showed that the sensitivity and specificity of CXR for the diagnosis of PTX were 82% (95% CI: 72–90%) and 96% (95% CI: 90–99%), whereas the sensitivity and specificity of LUS were 98% (95% CI: 93–100%) and 100% (95% CI: 96–100%). The authors showed that the lung point is not observed in severe PTX because of the large quantity of air beneath the ultrasound probe; this situation could be a limitation of LUS. However, the generalizability of the results is not completely acceptable since eligible studies included infants born at 31–33 weeks of gestation, and there was some heterogeneity about LUS techniques and equipment.

Montero Gato et al. [29] conducted an observational prospective investigation spanning from January 2018 to December 2020. During this period, lung ultrasound assessments were carried out as part of routine examinations of asymptomatic neonates admitted to the maternity ward. Out of the total 204 asymptomatic neonates enrolled in the study, 21 (10.3%) exhibited ultrasound indications suggestive of pneumothorax (Group A), while 183 (89.7%) showed normal lung ultrasound findings (Group B). On average, lung ultrasound examinations were conducted after 19 h of life (range 9–34 h). Notably, the presence of A-lines behind the sternum in the anterior transverse plane, specifically at the intermammillary level, was observed in 100% of patients in Group A, contrasting with the absence of such findings in Group B (*p* < 0.0001). Additionally, neonates in Group A displayed the lung point positioned along the midclavicular line, indicative of a minor air leak. The application of lung ultrasound enabled the precise identification of suspected small-sized air leaks, even in asymptomatic neonates. Consequently, the actual incidence of pneumothorax among asymptomatic neonates may surpass previous estimates reported in the literature. The clinical implications of these findings are unclear.

Montero Gato et al. [30] conducted a study to assess the utility of the central and anterior transverse thoracic planes in ultrasound diagnosis of pneumothorax, examine the diagnostic value of the “mirrored ribs” sign, and evaluate the predictive value of lung point localization for pleural drainage necessity. The study encompassed patients born between January 2014 and December 2020. Two groups of neonates admitted to the intensive care unit (ICU) due to respiratory distress were analyzed: Group A comprised 264 neonates with ultrasound-diagnosed PTX, with or without other pulmonary conditions (neonatal RDS, pneumonia, or meconium aspiration), while Group B (control) included 168 neonates admitted for respiratory distress without PTX detected via lung ultrasound. In both groups, images of the anterior longitudinal plane were retrospectively reviewed to identify pneumothorax signs and mirrored ribs. The mirrored ribs sign in the anterior longitudinal plane showed low diagnostic utility, as it was observed in 35.6% of patients with pneumothorax and in 36.9% of controls (*p* = 0.1505]. To evaluate the lung point’s utility in predicting pleural drainage necessity in Group A, 39 newborns with pneumothorax requiring drainage were compared with 272 newborns with non-drained pneumothorax. Lung point location significantly correlated with severe pneumothorax and the need for drainage (*p* < 0.001), with a PPV of 86.1% and an NPV of 97.1%. A-lines behind the sternum in the anterior transverse plane were present in 98.9% of newborns with pneumothorax compared to none in the controls (*p* < 0.001), demonstrating high sensitivity, specificity, and reproducibility for pneumothorax diagnosis. Patients with severe pneumothorax on lung ultrasound were more likely to require thoracic drainage. The limitations of this study are connected with its retrospective design and with the variability of operator experience and device settings, and the generalizability of the results is therefore reduced.

BPD, also known as chronic lung disease, is a frequent complication of prematurity. BPD is a clinical syndrome of lung injury that disrupts alveolarization and microvascular development, resulting in abnormal gas exchange and lung mechanics [4]. Pezza et al. [31] conducted a systematic review and meta-analysis to assess if early LUS (performed within the first 2 weeks of life) can accurately predict BPD development among infants born < 32 weeks of gestation. They included eight studies with 1027 infants. Overall, for the BPD prediction in the first 2 weeks of life, LUS has a pooled sensitivity and specificity ranging between 70% and 80% and between 80% and 87%, respectively. Since several factors (including but not limited to antenatal steroids, gender, gestational age at birth, and duration of invasive ventilation) may influence the occurrence of BPD and represent effect confounders, the authors planned to perform meta-regression analyses. They reported that prenatal steroid prophylaxis and gender do not significantly influence the diagnostic accuracy of LUS in predicting BPD or moderate/severe BPD. Meta-regressions for gestational age and duration of invasive ventilation were not performed for technical reasons. The authors compared a less extensive LUS score with a more extensive LUS score and found no significant differences between the two in predicting BPD; hence, they advocated the adoption of a less extensive LUS score for BPD prediction. The validity and generalizability of the results are good, even if BPD is a multifactorial spectrum rather than a simple disease, and the potential effects of several factors (including fetal growth restriction, gestational age, duration and type of invasive ventilation, surfactant administration modalities, among others) were not assessed by the authors.

Xu et al. [32] compared the predictive role of LUS and CXR in preterm infants. They conducted a study with 248 eligible infants, of which 80 were randomly enrolled and divided into a BPD group (*n* = 12) and a non-BPD group (*n* = 62); they showed that the diagnostic accuracy of lung ultrasound for bronchopulmonary dysplasia was better than that of X-rays (98.7% vs. 85.1%). The limitations of the study included the unclear study design, the interobserver variability, and the small sample size.

Li et al. [33] conducted a prospective observational study to assess the predictive value of LUS for the development of moderate-to-severe BPD (msBPD) at different time points in preterm infants born < 32 weeks of gestation. Out of 190 eligible infants in the study period, 150 were enrolled (63 with moderate-to-severe BPD and 87 with no or mild BPD). LUS scores were compared between groups using repeated measures analysis of variance. The authors reported that an early (in the first 2 weeks) LUS score predicted BPD and msBPD with 72.9% sensitivity and 90.7% specificity. The predictive performance of LUSs for msBPD was evaluated according to different GA groups (23–27 weeks and 28–32 weeks). The LUS score had lower diagnostic accuracy in infants born before 28 weeks (which are at higher risk of BPD) compared to those born after 28 weeks. The limitations of the study that affect its generalizability include the single-center design—and therefore potential differences in clinical practice—and the small proportion of infants born before 28 weeks who are at highest risk for BPD.

Sun and colleagues [34] performed a prospective study with 128 infants with gestational age < 34 weeks, including 30 without BPD, 31 with mild BPD, 23 with moderate BPD, and 44 with severe BPD, to investigate the role of a modified LUS score in predicting BPD. They showed that the modified LUS score at 36 weeks postmenstrual age (an extended LUS score analyzing two additional retrodiaphragmatic, retroepatic, and retrosplenic areas with a score ranging from 0 to 24) correlates significantly with short-term clinical outcomes (BPD severity and oxygen requirement at discharge). The ROC analysis of the modified LUS score also showed a significant advantage over the classic LUS score. The optimal cutoff points were 14 for moderate and severe BPD prediction and 16 for severe BPD prediction. This was in contrast to the results of a previous meta-analysis [31]. The limitations of this study included the use of a previous BPD definition and the inclusion of relatively mature infants who are at lower risk of BPD compared to extremely preterm infants.

To overcome this limitation, Zong et al. [35] conducted a single-center prospective observational study in infants born at or before 25 weeks of gestation to verify if the modified LUS score has good reliability to predict BPD and moderate-severe BPD (msBPD) and to test whether examination of posterior pulmonary fields could enhance diagnostic accuracy. A total of 89 infants were eligible; 64 subjects (72%) developed BPD, and 26 (29% of the total sample) had msBPD. Those who developed BPD and msBPD had a higher LUS score using anterolateral and posterior fields than those who did not (*p* < 0.0001). The AUC of the LUS score was 0.90 (95% CI: (0.83–0.97) *p* < 0.001) and 0.92 (95% CI: (0.85–1.00), *p* < 0.001) for the ability to predict any grade of BPD and msBPD. LUS was performed on the 14th day of life by an experienced sonographer blind to clinicians, while two independent evaluators blinded to clinical details took charge of evaluating the entire image. The limitations of this study included the single-center design, the assessment at a single timepoint, and the lack of inclusion of infants at 26–28 weeks of gestation.

Radulova et al. [36] conducted a single-center, prospective trial to describe the changes in ultrasound images as well as the appearance of lung consolidations (LC) in 124 very low-birth-weight infants with and without BPD. The LUS score was calculated on the seventh day of life, and posterior fields were excluded when scores were calculated. The number of LC in the non-BPD group was significantly lower (0–5) compared with the moderate–severe BPD group (3–45) from birth until the 36 weeks of GA. LUS scores were associated with severe BPD, and the calculation of the scores on the seventh day of life could be an excellent predictor of moderate–severe BPD. The authors did not provide details about the size of the LC and their potential association with mean airway pressure levels (and potential adjustments).

Shen et al. [37] conducted a prospective cohort study to assess the accuracy of LUS in predicting late respiratory outcomes within the first 2 years of life. They hypothesized that a modified LUS score (mLUS) could be more reliable than a conventional LUS score (cLUS) to predict late respiratory disease during the first 2 years of life in preterm infants. BPD status was assessed at 28 days postnatally, and severity was assessed at 36 weeks’ PMA using the modified NICHD BPD classification. Both the mLUS score and the cLUS score were significantly higher among infants with late respiratory disease compared to those without late respiratory disease (*p* < 0.001 for both). The AUC for the mLUS score was significantly higher than that for the cLUS score (*p* = 0.02). However, there was no significant difference in AUC for the modified NICHD BPD classification with either te mLUS score (*p* = 0.91) or the cLUS score (*p* = 0.28). The limitations of the study include the small sample size and the need for two different probes to calculate the mLUS score, which limit the generalizability of the results.

Neonatal pneumonia is one of the most common infectious diseases in neonates and may be associated with prolonged invasive ventilation and chronic lung disease [38]. Liu et al. [39] conducted a prospective observational study (40 infants with severe pneumonia compared to 40 infants without lung disease) to evaluate the diagnostic accuracy of LUS for neonatal pneumonia. All infants with pneumonia (but none in the control group) underwent CXR. The authors described LUS features of pneumonia (pleural line abnormalities, lung consolidation, interstitial syndrome, disappearance of lung sliding, lung pulse), and found that large areas of lung consolidation with irregular margins were 100% sensitive and 100% specific for the diagnosis of severe neonatal pneumonia. However, the study only included patients with severe pneumonia based on clinical criteria and did not report LUS findings in patients with mild pneumonia. Moreover, the sample size was small, and the study population included preterm and term infants; details (such as microbiology findings, clinical course of patients, ventilator modality) were not provided. Therefore, the generalizability of these findings to milder forms of pneumonia or to specific agents (i.e., viral versus bacterial pneumonia) is limited.

Ventilator-associated pneumonia (VAP) is a frequent cause of antibiotic administration and is associated with the development of BPD, prolonged mechanical ventilation, and hospital stays [40]. The definition of VAP among infants is challenging, with no international consensus, although usually based on CXR changes and clinical and laboratory findings. Tusor et al. [40] performed a quality improvement study to define VAP and evaluate a multiparameter score for VAP diagnosis that includes LUS features in preterm infants. They included 28 patients (56 VAP episodes) and provided details about clinical, laboratory, and CXR data. They built a post hoc multiparameter score for VAP diagnosis that included clinical, microbiology, and LUS features that was tested on days 1 and 3 of the onset of clinical symptoms. They found that adding LUS data to clinical information improves the predictive value of the VAP score as opposed to combining clinical information with CXR. A VAP score of >4 on Day 1 and >5 on Day 3 gave the highest sensitivity (0.94) with an AUC of 0.91 (95% CI 0.8–1.00) and 0.97 (95% CI 0.92–1.00), respectively. Sensitivity and specificity using CXR were 0.81 and 0.66, respectively.

Jiang et al. [41] conducted a study to compare CXR and LUS in 115 patients with ventilator-associated pneumonia (VAP). LUS findings of the confirmed cases showed a lung consolidation with an air bronchogram sign (111/115), alveolar-interstitial syndrome (113/115), pleural effusion (12/115), pleural line abnormalities (114/115), and lung pulse (15/115). CXR confirmed 109 cases of pneumonia. Taking the clinical diagnosis of VAP as the gold standard, the lung consolidation with air bronchogram sign on LUS had a higher sensitivity, specificity, and accuracy for the diagnosis of VAP than other LUS or CXR findings, with better consistency with the clinical diagnosis (AUC = 0.983, *p* < 0.05). Lung pulse was a very specific LUS sign of VAP. The limitations of the study were the lack of a description of clinical outcomes and the small sample size.

Dong et al. [42] conducted a systematic review and meta-analysis to assess the performance of LUS to diagnose pneumonia in children under 16 years of age. From a total of 252 articles, 26 observational studies (22 prospective and 4 retrospective) were analyzed. LUS has a high sensitivity (95%) and specificity (94%) in detecting VAP. Among the 26 studies, in 15 studies, LUS was conducted in pediatric emergency departments. Of the remaining 11 studies, eight were conducted in the pediatric ward, two in the NICU, and one in the PICU. Thus, the role of LUS in the diagnosis of VAP in neonates remains to be confirmed.

Meconium aspiration syndrome (MAS) is a rare and life-threatening disease caused by multiple pathophysiological mechanisms induced by meconium in lung tissue and airways [43]. In a prospective observational study, Liu et al. [44] assessed the diagnostic value of LUS for MAS. They studied 117 infants with MAS and 100 controls and found that lung consolidation with irregular margins was a common and specific ultrasound finding of MAS, with 100% diagnostic sensitivity and specificity. They suggested that MAS and pneumonia may be differentiated because varying degrees of pulmonary consolidation are usually observed in both lungs in MAS but only in one lung in pneumonia. However, details about the clinical characteristics of patients, including ventilator support and outcomes, were not provided, which limited the generalizability of the study findings.

Pulmonary hemorrhage of the newborn (PH) is a multifactorial critical disease often occurring in the first days of life and resulting in a high rate of complications and mortality [45]. Perinatal factors such as severe intrauterine pneumonia, birth asphyxia, RDS, and coagulation disorders have been proposed [45]. In a cohort study, Ren and colleagues [46] evaluated the diagnostic accuracy of LUS for PH. They reported that, among other LUS features, the visualization of shreds at the edge of the consolidation area (the shred sign) had a sensitivity of 91.2% and a specificity of 100% in diagnosing PH and is also useful for serial evaluation of the lesions. The limitations of this study include the unclear study design (prospective or retrospective) and patient selection, as well as the lack of clinical data about the patients. Therefore, the generalizability and validity of the results are limited. In a retrospective case–control study, Liu et al. [47] tried to identify specific echographic signs of PH in order to better diagnose neonatal PH by using LUS. They found that the detection of lung consolidation with fluid bronchograms and pleural effusion is a specific sign of PH, with a sensitivity of 81.0% and a specificity of 98.4%. The limitations of this study are due to the inclusion of non-consecutive patients, which may not be fully representative of all PH patients.

More recent applications of LUS in newborns include the prediction of extubation success and the assessment of diaphragm activity. In fact, invasive mechanical ventilation (MV) leads to the rapid development of diaphragmatic weakness due to the onset of muscle atrophy and contractile dysfunction. This condition has been defined as ventilator-induced diaphragmatic dysfunction and has been associated with extubation failure [48,49]. Other authors reported that the LUS score but not diaphragmatic excursion parameters could predict extubation failure [50]. According to a recent meta-analysis, LUS had good sensitivity and specificity in predicting extubation failure; however, the level of evidence and the methodological heterogeneity observed preclude firm conclusions [51].

The results and the quality of included articles were evaluated according to the Critical Appraisal Skills Programme (CASP) guidelines, available at https://casp-uk.net/casp-tools-checklists/, accessed on 12 February 2024, as shown in Supplementary Table S1.

**Figure 2:**
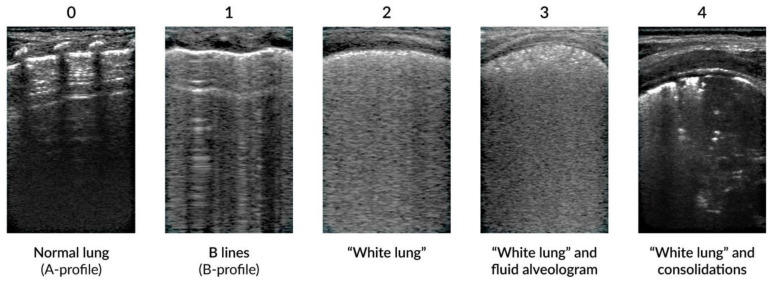
Frequent LUS findings in various respiratory conditions of the newborn (adapted from [21]).

Examples of LUS findings are shown in Figure 2. Most neonatal diseases consist of various combinations of these patterns; however, the examination of moving structures (i.e., pleura, diaphragm) is required in order to interpret the findings in light of the clinical picture of patients.

## 4. Discussion

This review showed that, according to the available evidence, LUS is a useful tool for the management of several neonatal respiratory conditions. The validity and generalizability of the results of the studies are moderate to good. In particular, according to the available evidence, LUS has good diagnostic accuracy for the most frequent diseases (RDS, TTN, PTX, MAS, pneumonia/VAP, pulmonary hemorrhage).

RDS is one of the most frequent neonatal respiratory conditions. LUS utility has been demonstrated in several studies with regard to RDS diagnosis (and differential diagnosis with other conditions), monitoring of respiratory status, indications for surfactant administration, lung recruitment, prediction of NIV failure, and extubation success. The body of evidence is growing and includes randomized trials and meta-analyses. However, there are limitations for the widespread use of LUS, such as the heterogeneity of studies regarding methods (i.e., probe, technique, operator skills), different methods to calculate LUS scores, differences in patient characteristics, and neonatal practice.

In particular, the utility of LUS for RDS diagnosis has been demonstrated by a meta-analysis with a high grade of heterogeneity [14] and a relatively small retrospective study [16]; hence, large, well-designed studies will be needed to confirm the role of LUS. Similarly, the utility of LUS in predicting MV and surfactant need among infants with RDS has been confirmed by a meta-analysis [10] and other studies, which mostly included infants with specific gestational age intervals [10,15,17,18,19]. Newer, interesting applications of LUS among infants with RDS that need further confirmation studies include lung recruitment guidance [20], outcome prediction [21], and monitoring of respiratory status [22]. Indeed, these studies were small or did not report all relevant outcomes. Future research is expected in this area in order to significantly improve the outcomes of infants with RDS.

TTN is another frequent neonatal condition with a benign course that has to be differentiated from RDS in the first phases. The diagnostic value of LUS for the diagnosis of TTN and for patient monitoring has been demonstrated by some studies: one meta-analysis, which included heterogeneous studies, and three single-center studies. Importantly, LUS can be more convenient than conventional radiology, as it can be repeated to monitor patients and can protect patients from radiation exposure. However, LUS findings need to be interpreted together with the clinical characteristics of patients, as TTN is finally a clinical diagnosis. Moreover, the sensitivity and specificity of CXR for the diagnosis of TTN are very high. Hence, the role of LUS in the diagnosis and management of TTN is promising, but more research is needed to clarify this issue.

Neonatal PTX is a life-threatening condition that requires prompt diagnosis and treatment. To this end, the use of LUS appears to be more convenient than conventional radiology to enable faster diagnosis and more precise and safe treatment (i.e., LUS-guided needle drainage). It is important to note that LUS has to be used in conjunction with clinical evaluation, as its high sensitivity may lead to the diagnosis of PTX in asymptomatic patients that would just need close observation. We presented the results of a meta-analysis [28], which included relatively mature infants and heterogeneous studies and showed that LUS may be more convenient than CXR for PTX diagnosis, even if severe PTX is a limitation of the LUS technique. Two other studies were a prospective one [29], which showed that LUS may overdiagnose PTX in asymptomatic infants, and a retrospective study [30], in which the authors proposed the utility of specific LUS scans for the diagnosis of PTX. Hence, future studies are required to better understand the utility of LUS in different categories of infants (e.g., extremely preterm infants and term infants), with multiple scans, and in different clinical settings.

BPD is a frequent complication of prematurity that has been linked to adverse outcomes. The utility of LUS is mainly linked to the prediction of this condition and to the implementation of preventive measures and therapies such as steroid administration. The calculation of LUS scores and the number of lung consolidations in the first weeks of life appear to be important for the prediction of BPD. However, limitations of LUS in published studies include differences in adopted LUS scores and techniques, in patients’ characteristics, and in clinical practice; also, a clear advantage of LUS for the improvement of clinical outcomes has not been demonstrated yet. One of the presented studies was a meta-analysis [31], which showed the early LUS with a less extensive score has 70–80% sensitivity and 80–87% specificity in predicting BPD. Other studies, including prospective ones [32,33], confirmed this finding, even if a relatively low number of extremely preterm infants were enrolled; moreover, in these studies, LUS was more accurate than CXR for BPD prediction. Other studies confirmed the utility of LUS performed at 14 days of life in extremely preterm infants [35] and the role of lung consolidations detected in the first weeks of life for the prediction of BPD [36]. However, clinical details (e.g., about ventilatory management and possible adjustments when findings such as lung consolidations were found) were not reported; hence, future studies assessing these important topics are warranted. Furthermore, two studies from the same group of researchers [34,37] showed that a modified LUS score including retrohepatic and retrosplenic areas is useful to predict short- and long-term outcomes in the first years of life in infants with BPD, and this was in contrast with the meta-analysis by Pezza et al. [31], which advocated the adoption of simpler scores. Thus, future studies are needed to clarify the role of LUS in the diagnosis, prognosis, and management of infants at high risk of BPD.

Neonatal pneumonia is one of the most common infectious diseases in neonates and may be associated with prolonged invasive ventilation and chronic lung disease. LUS has been shown to be effective for the diagnosis of pneumonia and for patient monitoring during this condition, with an important advantage in reducing radiation exposure. However, LUS findings are non-specific from an aetiological point of view and need to be compared and associated with clinical and laboratory findings [52]. We found a meta-analysis assessing the role of LUS in diagnosing pneumonia in children < 16 years of age (a small number of infants included) [42]. Other smaller studies showed that LUS has excellent sensitivity and specificity for the diagnosis of severe pneumonia [39] and is more accurate than CXR for the diagnosis of VAP [41]. Moreover, some authors reported that adding LUS data to clinical features is more accurate than adding CXR data for the diagnosis of VAP [40]. Hence, further studies to assess the role of LUS in milder forms of pneumonia caused by different agents are needed.

Meconium aspiration syndrome and pulmonary hemorrhage are rarer conditions for which LUS can have an important diagnostic role. Regarding MAS, a prospective study [44] showed that LUS is useful to distinguish between MAS and pneumonia, even if patient details could be better provided in order to find out predictive factors and outcomes. PH is a rarer condition, for which two studies [46,47] illustrated the more frequent and specific LUS signs: the shred sign and the presence of lung consolidations with fluid bronchograms and pleural effusion. More data are needed to confirm the role of LUS in these diseases.

Finally, other applications of LUS are the study of diaphragm activity and the prediction of extubation success, which can be important for the care of ventilated infants. To this end, further research is expected to elucidate the role of LUS and/or diaphragm ultrasound in improving the outcomes of infants on mechanical ventilation.

Studies have shown that one of the most important advantages of LUS implementation in clinical practice is a significant reduction (by 20–30%) of radiation exposure [7,53].

Some authors stated that whenever LUS is consistent with clinical and laboratory findings, CXR might be avoided; furthermore, when clinical findings are uncertain but a classic LUS pattern is evident (i.e., lung parenchyma consolidation with branched air bronchogram), we should consider avoiding CXR, using lung US to monitor the effects of therapy [52]. Whenever there is a discrepancy between LUS and other findings, CXR or CT (in selected cases) should be performed.

Potential obstacles to LUS implementation include the local availability of ultrasound machines and probes, the need for training, the potential contrast with minimal handling practices in preterm infants, quality assurance, and a potentially difficult initial learning process. In particular, for extremely preterm infants, the balance between pros and cons needs to be confirmed. Moreover, LUS features often need to be correlated with clinical and laboratory findings and cannot replace clinical evaluation, CXR, or CT scans. Finally, the standardization of scanning methods and parameter adjustment on ultrasound machines are needed to improve interobserver consistency, diagnostic accuracy, reliability of the method, and patient outcomes [54].

Future directions: the advantages of LUS should be confirmed in high quality studies with homogeneous characteristics of patients and techniques and in the most vulnerable patients, such as extremely preterm infants. Novel applications of LUS, such as those connected to the respiratory care of patients, need to be explored and confirmed.

## 5. Conclusions

Our review highlights the advantages and potential limitations of LUS for the diagnosis and treatment of the most frequent neonatal respiratory conditions. A growing body of evidence shows that LUS may have significant advantages over conventional radiology in terms of diagnostic accuracy for some conditions and reduction of radiation exposure. However, LUS needs to be used in conjunction with the clinical evaluation of patients and has some limitations, mainly connected to heterogeneity in techniques, operator dependence, and the clinical characteristics of patients included in published studies. Comparative studies to directly compare LUS with other diagnostic techniques in a clinical setting are needed.

## Data Availability

Not applicable.

## Supplementary Materials

The following supporting information can be downloaded at: https://www.mdpi.com/article/10.3390/jcm13113107/s1, Table S1: appraisal of selected studies.
