# Supplementary material for: Diagnostic Accuracy of Lung Ultrasound in Neonatal Diseases: A Systematized Review
Source: J Clin Med. 2024 May 25;13(11):3107. doi: 10.3390/jcm13113107 (PMC11172746; doi:10.3390/jcm13113107)
Supplement: Supplementary file 1 [file jcm-13-03107-s001.zip › jcm-2955948-supplementary.pdf]

Table S1: appraisal of selected studies.

1. Systematic Reviews

	**1) Did the review address a clearly focused question?**	**2) Did the authors look for the right type of papers?**	**3) Do you think all the important, relevant studies were included?**	**4) Did the review's authors do enough to assess quality of the included studies?**	**5) If the results of the review have been combined, was it reasonable to do so?**	**6) What are the overall results of the review?**	**7) How precise are the results?**	**8) Can the results be applied to the local population?**	**9) Were all important outcomes considered?**	**10) Are the benefits worth the harms and costs?**
Ma 2020a (RDS)	Yes	Yes	Yes	Yes	The authors performed subgroup analyses due to heterogeneity	LUS sensitivity and specificity were higher than CXR, particularly with transthoracic approach	Results are precisely shown, but heterogeneity must be taken into account	To preterm infants. More information needed (i.e. antenatal steroids, delivery mode, reasons for preterm birth, foetal growth restriction etc.)	Yes	Yes
Razak 2020 (surfactant need during RDS)	Yes	Yes	Yes	Yes	Yes	LUS score >5-6 had 88% (95% CI 80% to 93%) sensitivity and 82% (95% CI 74% to 89%) specificity for surfactant treatment	Very precise	To preterm infants born < 34 weeks of gestation	Yes	Yes

Luo 2023 (RDS)	Yes	Yes	Yes	Yes	The authors performed subgroup analyses due to heterogeneity	Sensitivity and specificity of LUS in predicting surfactant use were 86% (95% CI 81% to 90) and 82% (95% CI 71% to 90%). LUS within 1–3 h after birth had 89% sensitivity (95% CI 79% to 95%); LUS score ≤ 5 had 94% of sensitivity (95% CI 85% to 97%) in predicting surfactant use.	Results are precisely shown, but heterogeneity must be taken into account	To some extent	Yes	Yes
Ma 2020b (TTN)	Yes	Yes	Yes	Yes	The authors performed subgroup analyses due to heterogeneity	LUS sensitivity and specificity were 96% and 98% (cohort studies)	Results are precisely shown, but heterogeneity must be taken into account	Infants were born at 30-38 weeks. More information needed (i.e. antenatal steroids, delivery mode, reasons for preterm birth, foetal growth restriction etc.)	Yes	Yes

Wang 2022 (TTN)	Yes	Yes	Yes	Yes	The authors performed subgroup analyses due to heterogeneity	LUS is a promising method to diagnose TTN.	Very precise	Not defined	Yes	Yes
Fei 2021 (PTX)	Yes	Yes	Yes	Yes	Yes	Better diagnostic accuracy for LUS vs. CXR	Very precise	To infants born at 31-33 weeks of gestation More information needed (i.e. antenatal steroids, delivery mode, reasons for preterm birth, foetal growth restriction etc.)	Yes	Severe PTX can be challenging for LUS
Pezza 2022 (BPD)	Yes	Yes	Yes	Yes	The authors performed meta-regression to explore the effects of LUS and other factors for early BPD prediction	LUS sensitivity for the diagnosis of BPD was 70-80%, specificity 80-87%.	Results are precisely shown, but heterogeneity and lack of assessment of several factors limit the generalisability and validity of the results.	To some extent	Yes	Yes
Dong 2023 (pneumonia)	yes	yes	yes	yes	The authors performed meta-analysis to explore the performance of	LUS has a high sensitivity (95%) and specificity (94%).	Very precise	Partially (the study included children under 16 years of age).	yes	yes

					thoracic ultrasound in diagnosing pneumonia.					
Mohsen 2023 (extubation)	Yes	Yes	Yes	Yes	Yes	The pooled sensitivity and specificity for LUS in predicting extubation failure in neonates were 0.82 (95% CI: 0.75–0.88) and 0.83 (95% CI: 0.78–0.86), respectively.	Very precise	Yes	Yes	Yes

2. Randomised Controlled Trials

	**1) Did the study address a clearly focused research question?**	**2) Was the assignment of patients to interventions randomised?**	**3) Were all participants who entered the trial accounted for at its conclusion?**	**4) Were participants, investigators and study personnel 'blind' to intervention?**	**5) Were the study groups similar at the start of the randomised controlled trial?**	**6) Apart from the experimental intervention, did each study group receive the same level of care (that is, were they treated equally)?**	**7) Were the effects of intervention reported comprehensively?**	**8) Was the precision of the estimate of the intervention or treatment effect reported?**	**9) Do the benefits of the experimental intervention outweigh the harms and costs?**

Abushady 2021 (lung recruitment during RDS)	Yes	Yes	Yes	Investigators could not be blinded to the intervention	Yes	Yes	Yes, for short-term outcomes	Yes	Yes (in terms of oxygen and ventilation requirement, NICU stay)

3. Cohort studies

	1) Did the study address a clearly focused issue?	2) Was the cohort recruited in an acceptable way?	3) Was the exposure accurately measured to minimise bias?	4) Was the outcome accurately measured to minimise bias?	5) (a) Have the authors identified all important confounding factors?	5) (b) Have they taken account of the confounding factors in the design and/or analysis?	6) (a) Was the follow up of subjects complete enough?	6) (b) Was the follow up of subjects long enough?	7) What are the results of this study?	8) How precise are the results?	9) Do you believe the results?	10) Can the results be applied to the local population?	11) Do the results of this study fit with other available evidence?	12) What are the implications of this study for practice?
Corsini 2023 (RDS)	Yes	Partially (convenience sample)	Yes	Yes	Yes	Yes	Yes (for short-term outcomes)	Yes (for short-term outcomes)	Brat, Raimondi, and Rodriguez-Fanjul scores have all showed a strong ability to predict the	Precise	Yes	Yes	Yes	Brat, Raimondi, and Rodriguez-Fanjul scores have all a strong ability to predict the need for

									need for surfactant: AUCs 85% (95% CI 74% to 96%), 85% (95% CI 75% to 96%), and 79% (95% CI 67% to 92%), respectively.					surfactant with no significant differences
Perri 2022 (RDS)	Yes	Yes	Yes	Yes	Yes	Yes	Yes (for short-term outcomes)	Yes (for short-term outcomes)	LUS is useful to predict the need for CPAP with an AUC of 91% within 3 h of life and 82% at 4–6 h of life.	Precise	Yes	Yes	Yes	An early LUS score is a good predictor of the need for respiratory assistance with CPAP and surfactant administration in newborns with GA ≥ 33 weeks. LUS is also significant

														ly correlated with SpO2/FiO2 ratio.
VC Kumar 2024 (RDS)	Yes	Yes	Yes	Yes	Yes	Yes	Yes (for short-term outcomes)	Yes (for short-term outcomes)	LUS cutoff > 7 predicts NIV failure with a 77.4% sensitivity (95% CI: 58.9% to 90.8%), 75.1% specificity (95% CI 67.8% to 81.7%) and 75.5% overall accuracy (95% CI 68.8% to 81.4%).	Precise	Yes	Partially due to lack of data	Yes	LUS cutoff of>7 is useful in determining invasive ventilation needs during the initial 72 h of NIV.

Raimondi 1 2021 (RDS)	Yes	Yes	Yes	Yes	Yes	Yes	Yes (for short-term outcomes)	Yes (for short-term outcomes)	Inverse relation between LUS and SatO2/FIO2 ratio. LUS is also an early predictor of BPD especially in the 28 to 30 weeks' GA cohort with an AUC of 89%, cutoff of 10, sensitivity of 78% and specificity of 87%.	Precise	Yes	Yes	Yes	In preterm neonates affected by RDS, LUS is gestational age dependent, significantly correlates with the oxygenation status, and allows for an early bronchopulmonary dysplasia prediction.
Raimondi 2 2021 (RDS)	Yes	Yes	Yes	Yes	Yes	Yes	Yes (for short-term outcomes)	Yes (for short-term outcomes)	LUS predicted, without significant difference among different	Precise	Yes	Yes	Yes	LUS is a valid criterion to administer the first surfactant dose

									gestational age groups, the first surfactant administration with AUC of 86% (95% CI 81 to 91%), cut off of 9, sensitivity of 79% (95% CI, 70% to 86%), specificity of 83% (95% CI, 76% to 89%).					regardless of GA
Szymański 2023 (RDS)	Yes	Partially (infants who were primarily intubated were excluded)	Yes	Yes	Partially	Partially	Yes (for short-term outcomes)	Yes (for short-term outcomes)	LUS has significant predictive value regard to critical	Not much	Yes	Partially	Yes	LUS is useful to predict important outcomes in neonatal RDS.

									outcomes in neonatal RDS.					
Copetti 2007 (TTN)	Yes	Yes	Yes	Yes	Partly	Partly	Yes	Yes	Sensitivity and specificity of the double lung point was 100% for the diagnosis of TTN.	Yes	Yes	Yes	Yes	LUS could become the first approach for imaging of neonatal respiratory distress.
Srinivasan 2022 (TTN)	Yes	Yes	Yes	Yes	Yes	Yes	Yes	Yes	Pulmonary edema, double lung point and coalescent B lines in the absence of consolidation has 100% sensitivity and specificity	Enough	Yes	Yes	Yes	Lung ultrasound can be thus used as an initial screening tool in neonatal intensive care units for lung diseases.

									in diagnosing TTN.					
Pezza 2023 (TTN)	Yes	Yes	Yes	Yes	Partially (lack of data)	Yes	Yes	Yes	The RDS cohort had worse lung aeration and oxygenation compared with the TTN cohort in the first 72 hours of life.	Good	Yes	Yes	Yes	Lung aeration and function can be estimated by LUS in the first 72 hours of life.
Gregorio – Hernàndez 2022 (PTX)	Yes	Yes (lack of sample)	Yes	Yes	Yes	Yes	Yes	Yes	LUS is a straightforward, rapid, uncomplicated, and precise tool for assessing newborns with NP,	Yes	Yes	Yes	Yes	Lus is the best way to to evaluating newborns with NP

									including those in typical positions such as surgical patients.					
Montero Gato 2023 (PTX)	Yes	Yes	Yes	Yes	Yes	Yes	Yes	Yes	LUS showed evidence of PTX in around 10% of asymptomatic infants.	Yes	Yes	Yes	Yes	the actual incidence of pneumothorax among asymptomatic neonates may surpass previous estimates reported in the literature.
Montero Gato 2022 (PTX)	Yes	Yes	Yes	Yes	Yes	Yes	Yes	Yes	A-lines in the anterior transverse plane	Yes	Yes	Yes	Yes	The mirrored ribs sign showed low diagnostic

									present high sensitivity, specificity, and reproducibility for the diagnosis of PTX.					utility for neonatal PTX.
Shen 2023 (BPD)	Yes	Yes	Yes	Yes	Yes	Yes	Yes	Yes	The predictive accuracy of the modified LUS score for late respiratory disease was not inferior to the modified NICHD-defined BPD classificati	Precise	Yes	Yes	Could not find similar studies about BPD.	mLUS score at 36 weeks' PMA for preterm infants with gestational age < 32 weeks significantly correlates with late respiratory disease status.

									on, and it was significantly superior to the classic LUS scores. The AUCs were 0.820 for the mLUS score , 0.825 for modified NICHD-defined BPD classification, 0.776 for the cLUS score.					
Radulova 2022 (BPD)	Yes	Yes	Yes	Yes	Partially	Partially	Yes (for short-term outcomes)	Yes (for short-term outcomes)	The number of lung consolidations in the non-BPD	Very precise	Yes	Yes	Could not find similar studies about BPD.	Calculation of the scores on the seventh DOL could be an excellent

									group was significantly lower (0-5) compared with the moderate–severe BPD group (3–45) ($p < 0.05$) and the difference was significant for the period between 1 week and 1 month after birth ($p = 0.001$).					predictor of moderate–severe BPD.
Zong 2023 (BPD)	Yes	Yes	Yes	Yes	Yes	Yes	Yes (for short-term outcomes)	Yes (for short-term outcomes)	The LUS score-anterolateral on the 14th day of	Precise	Yes	Yes	Yes	The LUS score on the 14th day of life can be a

									life showed a moderate diagnostic accuracy to predict BPD and msBPD (AUC 0.797 and 0.811, respectively), while the LUS score-anterolateral combined with posterior significantly improved diagnostic accuracy of BPD and msBPD (AUC					useful tool to predict the development of any grade BPD and msBPD in preterm infants with a gestational age ≤ 25 weeks.

									0.902 and 0.922 respectively).					
Sun 2022 (BPD)	Yes	Yes	Yes	Yes	Yes	Yes	Yes (for short-term outcomes)	Yes (for short-term outcomes)	The modified LUS score significantly correlated with all the short-term clinical outcomes adjusted for gestational age. The classic LUS scores were also associated with most outcomes, except for oxygen requirement at	Very precise	Yes	yes	Yes	Lung ultrasound can accurately and noninvasively assess the severity of BPD at 36 weeks of PMA and evaluate short-term clinical outcomes.

									discharge and postnatal systemic steroids treatment, but the correlation was weaker than mLUS scores.					
Li 2023 (BPD)	Yes	Partially	Yes	Yes	Partially (lack of data)	Partially	Yes (for short-term outcomes)	Yes (for short-term outcomes)	Early LUS has 72.9% sensitivity and 90.7% specificity for BPD prediction. LUS seems to be less helpful in infants born before 28 weeks.	Precise	Yes	yes	Could not find similar studies about BPD	Early LUS, in the first 2 weeks of postnatal age, can predict BPD and msBPD in infants with GA < 32 weeks.

Xu 2023 (BPD)	Yes	Yes	Yes	Yes	Yes	Partially	Yes (for short-term outcomes)	Yes (for short-term outcomes)	The diagnostic accuracy of LUS to predict bronchopulmonary dysplasia is higher than chest-X ray (98.65% vs 85.14%).	Not much	Yes	Yes	Yes	LUS may be better than CXR to predict BPD.
Liu 2014 (Pneumonia)	Yes	Partially (convenience sample)	Yes	Yes	Partially (lack of data)	Partially	Yes (for short-term outcomes)	Yes (for short-term outcomes)	LUS features (large areas of lung consolidation with irregular margins) have sensitivity 100% and specificity 100% for severe	Not much	Yes	Partially due to lack of data.	Yes (LUS is widely used for pneumonia in children)	LUS is useful to detect severe pneumonia.

									pneumonia.					
Ma 2023 (pneumonia)	Yes	Yes	Yes	Yes	Yes	Yes	Yes (for short-term outcomes	Yes (for short-term outcomes)	LUS has a role in the diagnosis and judgment of the severity of neonatal infectious pneumonia, but it cannot be used for pathogenic identification in the early stages of pneumonia	Precise	Yes	No (The data did not satisfy a normal distribution or homogeneity of variances)	there is considerable controversy as to whether LUS can differentiate the etiology of pneumonia	there is no difference in the LUS signs of neonatal pneumonia between different pathogens, different infection times, or different gestational ages. The size and extent of pulmonary consolidation have high sensitivity and specificity for the differentiation of severe and mild

														neonatal pneumonia
Jiang 2022 (pneumonia)	Yes	Partially (convenience sample)	Yes	Yes	Yes	Partially	No	No	LUS combined with air bronchogram have higher sensitivity, specificity, and accuracy (96.5%, 100%, 96.7% respectively) for the diagnosis of VAP than CXR (94.8%, 71.4%, 93.4% respectively). The sensitivity and	Very precise	Yes	Partially (small sample size)	Could not find similar studies about VAP.	Lung consolidation with air bronchogram had a higher sensitivity, specificity, and accuracy for the diagnosis of VAP compared to CXR.

									specificity of LUS (94.7 and 89.6%,) in evaluating the weaning from the ventilator were higher than those of CXR (73.7 and 84.4%, respectively).					
Tusor 2021 (VAP)	Yes	Yes	Yes	Yes	Yes	Yes	Yes (for short-term outcomes)	Yes (for short-term outcomes, i.e. BPD at 36 weeks post-menstrual age)	Adding LUS to clinical information improves the predictive value of the VAP score as	Yes	Yes	Yes	Could not find similar studies about VAP.	Adding LUS to clinical information is an alternative to CXR to diagnose VAP.

									opposed to combining clinical nformation with CXR.					
Liu 2016 (MAS)	Yes	Partially (convenience sample)	Yes	Yes	Partially (lack of data)	Partially	Yes (for short-term outcomes)	Yes (for short-term outcomes)	LUS features (lung consolidation with irregular margins) have sensitivity 100% and specificity 100% for MAS	Not much	Yes	Partially due to lack of data	Yes (small case series)	LUS is useful to detect MAS
Ren 2017 (PH)	Yes	Partially (lack of details about recruitment of patients and controls)	Yes	Yes	Partially (lack of data)	Partially	Yes (for short-term outcomes)	Yes (for short-term outcomes)	LUS features (shred sign) have sensitivity 91.2% and specificity 100% for PHN	Not much	Yes	Partially due to lack of data	Yes	LUS is useful to detect PHN

Liu 2023 (PH)	Yes	Partially (inclusion of non-consecutive sample that may not be fully representative of all PH patients)	Yes	Yes	Yes	Yes	Yes (for short-term outcomes)	Yes (for short-term outcomes)	LUS features (lung consolidation with fluid bronchograms and pleural effusion) have sensitivity 81.0% and specificity 98.4% for PHN.	Not much	Yes	Partially	Yes	LUS is useful to detect PHN
Nobile 2024 (diaphragm atrophy)	Yes	Yes	Yes	Yes	Yes	Yes	Yes (for short-term outcomes)	Yes (for short-term outcomes)	Diaphragm atrophy, significantly associated with extubation failure, occurred in 58.8% of the study infants on	Precise	Yes	Partially (patients were very sick)	Could not find similar studies	Diaphragm ultrasound has a potential role to assess atrophy and predict extubation failure.

									endotracheal ventilation.					
Mohsen 2022 (extubation)	Yes	Yes	Yes	Yes	Yes	Yes (there were no local guidelines for extubation during the study period)	Yes (for short-term outcomes)	Yes (for short-term outcomes)	LUS was an independent predictor for successful extubation (odd ratio 0.46, 95% confidence interval [0.23–0.9], p = 0.02), and a cut-off value of ≥15 had 95% sensitivity and 85% specificity in detecting	Precise	Yes	Yes	Could not find similar studies	Lung, but not diaphragm ultrasound, has good accuracy for predicting successful extubation.

									extubation failure.					
